# The Effects of Oral Liposomal Glutathione and In Vitro Everolimus in Altering The Immune Responses Against *Mycobacterium bovis* BCG Strain in Individuals With Type 2 Diabetes

**DOI:** 10.1515/bmc-2021-0003

**Published:** 2021-05-09

**Authors:** Kimberly To, Ruoqiong Cao, Aram Yegiazaryan, James Owens, Kayvan Sasaninia, Charles Vaughn, Mohkam Singh, Edward Truong, Airani Sathananthan, Vishwanath Venketaraman

**Affiliations:** Graduate College of Biomedical Sciences, Western University of Health Sciences, Pomona, CA, USA; College of Osteopathic Medicine of the Pacific, Western University of Health Sciences, Pomona, CA, USA; Graduate College of Biomedical Sciences, Western University of Health Sciences, Pomona, CA, USA; College of Osteopathic Medicine of the Pacific, Western University of Health Sciences, Pomona, CA, USA; College of Osteopathic Medicine of the Pacific, Western University of Health Sciences, Pomona, CA, USA; Graduate College of Biomedical Sciences, Western University of Health Sciences, Pomona, CA, USA; Graduate College of Biomedical Sciences, Western University of Health Sciences, Pomona, CA, USA; Graduate College of Biomedical Sciences, Western University of Health Sciences, Pomona, CA, USA; Graduate College of Biomedical Sciences, Western University of Health Sciences, Pomona, CA, USA; College of Osteopathic Medicine of the Pacific, Western University of Health Sciences, Pomona, CA, USA; Graduate College of Biomedical Sciences, Western University of Health Sciences, Pomona, CA, USA; College of Osteopathic Medicine of the Pacific, Western University of Health Sciences, Pomona, CA, USA

**Keywords:** Glutathione, immune responses, mycobacteria, cytokines, host-directed therapies, everolimus, diabetes

## Abstract

Tuberculosis (TB) caused by *Mycobacterium tuberculosis* (*M. tb*) still remains a devastating infectious disease in the world. There has been a daunting increase in the incidence of Type 2 Diabetes Mellitus (T2DM) worldwide. T2DM patients are three times more vulnerable to *M. tb* infection compared to healthy individuals. TB-T2DM coincidence is a challenge for global health control. Despite some progress in the research, *M. tb* still has unexplored characteristics in successfully evading host defenses. The lengthy duration of treatment, the emergence of multi-drug-resistant strains and extensive-drug-resistant strains of *M. tb* have made TB treatment very challenging. Previously, we have tested the antimycobacterial effects of everolimus within *in vitro* granulomas generated from immune cells derived from peripheral blood of healthy subjects. However, the effectiveness of everolimus treatment against mycobacterial infection in individuals with T2DM is unknown. Furthermore, the effectiveness of the combination of *in vivo* glutathione (GSH) supplementation in individuals with T2DM along with *in vitro* treatment of isolated immune cells with everolimus against mycobacterial infection has never been tested. Therefore, we postulated that liposomal glutathione (L-GSH) and everolimus would offer great hope for developing adjunctive therapy for mycobacterial infection. L-GSH or placebo was administered to T2DM individuals orally for three months. Study subjects’ blood was drawn pre- and post- L-GSH/ or placebo supplementation, where Peripheral Blood Mononuclear Cells (PBMCs) were isolated from whole blood to conduct *in vitro* studies with everolimus. We found that *in vitro* treatment with everolimus, an mTOR (membrane target of rapamycin) inhibitor, significantly reduced intracellular *M. bovis* BCG infection alone and in conjunction with L-GSH supplementation. Furthermore, we found L-GSH supplementation coupled with *in vitro* everolimus treatment produced a greater effect in inhibiting the growth of intracellular *Mycobacterium bovis* BCG, than with the everolimus treatment alone. We also demonstrated the functions of L-GSH along with *in vitro* everolimus treatment in modulating the levels of cytokines such as IFN-γ, TNF-α, and IL-2 and IL-6, in favor of improving control of the mycobacterial infection. In summary, *in vitro* everolimus-treatment alone and in combination with oral L-GSH supplementation for three months in individuals with T2DM, was able to increase the levels of T-helper type 1 (Th1) cytokines IFN-γ, TNF-α, and IL-2 as well as enhance the abilities of granulomas from individuals with T2DM to improve control of a mycobacterial infection.

## Introduction

*Mycobacterium tuberculosis* (*M. tb*), the etiological agent for tuberculosis (TB) caused 1.5 million deaths and 10 million active cases of disease in the year 2018 [1]. According to the World Health Organization (WHO), 1.2 million deaths from the 1.5 million total were from HIV-negative people, and about 251,000 deaths were from HIV-positive people [1]. Additionally, it is estimated that about a quarter of the world’s population has latent TB (LTBI) [1]. Approximately, 5-10% of people with (LTBI) have risks for developing an active form of TB in their lifetime [1]. Despite the availability of anti-TB drugs and the attenuated BCG vaccine, there is no significant drop in the number of TB cases [1]. Immunocompromised individuals such as HIV patients, diabetics, those in the condition of undernutrition, smokers, and individuals with heavy alcohol consumption are more susceptible to an active TB infection [1]. HIV patients and individuals with type 2 diabetes (T2DM) are at 19 times and 3 times higher risk for developing active TB than healthy individuals, respectively [2]. Additionally, the worldwide increased prevalence of T2DM in low and middle-income countries is currently recognized as an emerging risk and challenge to control TB [2]. T2DM is characterized by hyperglycemia caused by insulin resistance and accounts for 90-95% of the total prevalence of diabetes [3].

There are no obvious, outward symptoms for latent TB cases, while the active TB symptoms include a cough, coughs with bloody sputum, fatigue, fever, weight loss, night sweats, and sometimes death if there is no medical intervention. The Center for Disease Control (CDC) recommends a treatment regimen for active TB that includes four antibiotics: Isoniazid (INH), Rifampin (RIF), Pyrazinamide (PZA), and Ethambutol (EMB) [4]. However, there are moderate adverse effects from using these antibiotics. For example, INH affects the liver function and sometimes causes fatal hepatitis [5]. Taking RIF can result in fever, gastrointestinal disorder, and other immunological reactions [6]. However, the most common side effect of the aforementioned antibiotics is hepatotoxicity, therefore, frequent liver function tests need to be applied during the lengthy administration of antibiotics [7]. The emergence of multidrug and extensively-drug resistant strains of *M. tb* challenge the traditional anti-TB treatment regimen as well [8]. Thus, novel therapeutic strategies are in need of being developed.

*M. tb* infection preliminarily infects alveolar macrophages. This occurs when *M. tb* is phagocytosed by macrophages. Unlike other infectious organisms purposefully evading the phagocytosis, *M. tb* uses its cell wall receptors to access macrophages and bypass their defenses [9]. *M. tb* promotes the maturation of the phagosomal compartments so that it favors its own survival in the macrophages [10,11]. Matured macrophages can initiate a cascade of events resulting in the formation of a tight, robust structure known as a granuloma [12]. A significant amount of pro and anti-inflammatory cytokines and chemokines are involved in this reaction. A balance of anti-inflammatory and pro-inflammatory cytokines plays an important role in maintaining the granuloma structure and *M. tb* infection containment [13]. At an early time of granuloma formation, TNF-α, which is produced by infected macrophages, plays a crucial role in modulating the production of other cytokines and the maintenance of the granuloma, as well as connects the innate and adaptive immune responses against *M. tb* infection [14,15]. In brief, T-helper type 1 ( Th1) cells, a subset of CD4 T cells, secrete IFN-γ, IL-2, and lymphotoxin A [16]. IFN-γ is essential in activating the macrophages to eradicate the mycobacteria intracellularly [16]. CD8 T cells and Natural Killer (NK) cells enhance mycobacterial killing by releasing antimicrobial peptides such as perforin and granulysin [17].

Immune responses play a pivotal role in fighting against a *M. tb* infection; however, it does not always have a robust response to *M. tb* clearance. One approach to combat *M. tb.* infection is to identify host-directed therapies (HDT) or strategies that can modulate specific host immune pathways for more effective killing of *M. tb* [8]. HDT for *M. tb.* aims to augment host immune response or metabolism to optimize the use of antibiotics for better clearance of *M. tb.* [8]. Since HDT targets host cell function, *M. tb.* cannot develop resistance against HDT and therefore, can diminish the emergence of drug-resistant strains of *M. tb* and possibly shorten treatment duration [18].

A potential HDT is glutathione (GSH), a biological antioxidant, recognized for maintaining redox homeostasis [19]. GSH is synthesized in every cell in higher eukaryotes, although cellular concentrations and turnover rates differ among cell and tissue types [20]. GSH is a tripeptide antioxidant made of glutamine, cysteine, and glycine [19]. Over the last few years, our laboratory has focused on understanding the roles of GSH in immune responses against *M. tb* infection. There are two forms of GSH: reduced GSH (rGSH) and oxidized GSH (GSSG). rGSH is the functional form, which contains antioxidant characteristics, preventing cellular damage via detoxifying reactive oxygen species (ROS) [19]. Once reacted with ROS, two molecules of rGSH will become GSSG and water [19].

Another potential HDT is everolimus, a small molecule that could modulate autophagy to enhance the killing of mycobacteria through inhibition of the membrane target of rapamycin (mTOR) pathway. Autophagy is a homeostatic cellular process that removes intracellular debris. It acts as a catabolic process that is activated when cells lack nutrients or under cellular damage and stress through degrading damaged organelles and misfolded or abnormal proteins [8]. Autophagy is also part of the innate immune system and can be activated by invading pathogens, acting as one of the macrophage defense mechanisms against *M. tb* [8].

Autophagy is regulated by multiple, complex networks that are either independent or dependent of mTOR. mTOR is a master regulatory molecule that participates in cell metabolism, growth, proliferation, translation initiation, and cytoskeletal organization [8]. mTOR inhibition has been observed to improve cell survival and enhance host cell mechanisms against invading pathogens [8]. Everolimus, a derivative of rapamycin, is mainly used as a chemotherapy drug for renal cell cancer and an immunosuppressant to prevent rejection of organ transplants. However, mTOR inhibition by everolimus has been shown to promote autophagy and improve the cellular immune response in human studies [21]. Rapamycin can also induce mTOR inhibition, but everolimus has better solubility, oral availability, and decreased mean elimination half-life than its parent compound (rapamycin). Additionally, everolimus has better absorption and higher bioavailability [8].

Our lab has previously shown that *in vitro* treatment of everolimus to *in vitro* human granulomas reduced the burden of Erdman strain of *M. tb* significantly and was efficacious in controlling the infection alongside anti-TB drugs [22]. In this same study, there were also higher levels of autophagy and inhibition observed in everolimus-treated *in vitro* generated granulomas [22]. Due to its immune-suppressing effects, more research is required to fine-tune the dosage of everolimus to ensure that it can be used at an immune-modulating dose to serve as an adjunct therapeutic molecule alongside the standard anti-TB drugs.

In this study, we conducted a clinical trial to determine the effects of oral liposomal glutathione (L-GSH) supplementation for three months in individuals with Type 2 diabetes (T2DM) in conjunction with *in vitro* everolimus in improving the granulomatous responses against *M. bovis* BCG infection. Our study findings indicate that *in vivo* administration of L-GSH for three months along with *in vitro* treatment with everolimus, an mTOR inhibitor, improved the ability of immune cells derived from individuals with T2DM to better control BCG infection.

## Material and Methods

### Study Design and Intervention

**Figure 1:**
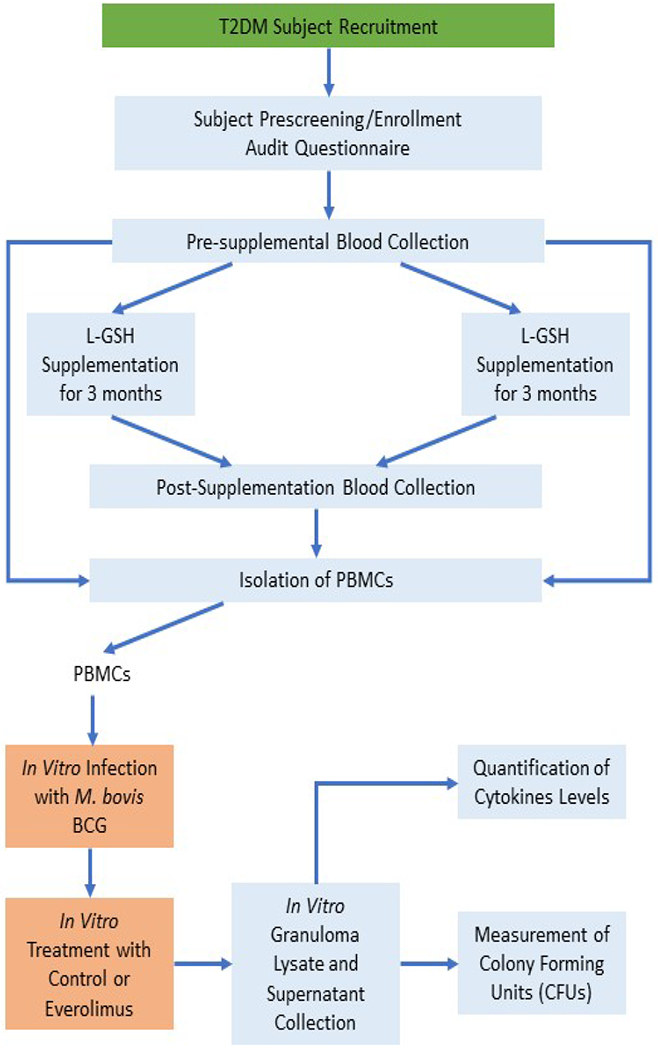
Schematic representation of the overall study design.

This research consisted of a double-blind, randomized, placebo-controlled, clinical trial conducted at Western University of Health Sciences (WesternU). The experimental and placebo groups of the study were each formed by randomizing subjects into each study group as they were enrolled. For the clinical trial, the experimental group took oral liposomal glutathione (L-GSH) for a three-month period. At the same time, the placebo group took oral empty liposomes (placebo) for a three-month period (Figure 1). All subjects in both groups took 1.5 teaspoons of oral liposomal glutathione or empty liposomes twice a day (morning and evening), for a sum amount of 3 teaspoons per day (15 ml or 1260 mg of L-GSH). Your Energy Systems manufactured the oral L-GSH supplement as ReadiSorb™ LRG, a dietary additive delivering liposomal, reduced GSH suspended in liquid. The ingredients of L-GSH are **a) Reduced l-glutathione: Approximately** 425 mg per teaspoon in a new bottle. This concentration can be gradually reduced over time due oxidization. At the end of its second-year shelf life, when stored in the refrigerator, there will be approximately 350 mg of reduced glutathione.b) **Purified water** c) **Lecithin (non-GMO):** The liposomes in ReadiSorb Glutathione are derived from non-GMO lecithin. The lecithin is an extract of non-GMO soy oil; there is no protein in this product d) **Glycerin:** Glycerin is used as a sweetener and preservative. It supports the stability of the liposomes and allows for the extended shelf life. Glycerin is a normal product of fat metabolism and is readily converted to glucose for metabolism. Our glycerin is derived from palm fruit oil d) **Potassium Sorbate:** A natural material used as a preservative to prevent yeast and mold growth. It is a form of sorbic acid, a naturally occurring fatty acid which is easily metabolized in the body. Potassium sorbate is used to preserve wine, baked goods and cheese.

Your Energy Systems also manufactured the placebo supplement with the same exact materials, but with empty liposomes. Every component in ReadiSorb™ LRG is natural and considered by the Food and Drug Administration (FDA) to be GRAS (Generally Recognized as Safe). No documented toxicity in children using LRG (Liposomal rGSH) daily for two months has been found [23]. Previous studies demonstrated that there were no adverse reactions associated with the use of LRG at the same dose daily for three months in HIV-positive subjects, subjects with full-blown AIDS (Acquired Immunodeficiency Syndrome), and healthy subjects [24,25]. Table 1 and Figure 1 show the in vivo and in vitro components in the clinical trial. For the in vivo portion, we determined the effects of L-GSH on the immune responses against M. bovis BCG by either giving T2DM subjects the L-GSH or placebo (empty liposomes) supplement. For the in vitro portion of the study, we examined the effects of everolimus treatment (1 nM) against M. bovis BCG infection using PBMCs isolated from the patients in the GSH clinical trial.

#### Informed consent:

Informed consent has been obtained from all individuals included in this study.

#### Ethical approval:

The research related to human use has been complied with all the relevant national regulations, institutional policies and in accordance the tenets of the Helsinki Declaration, and has been approved by WesternU Institutional Review Board (IRB); protocol #FB17/IRB/031.

### Subject Selection, Clinical Encounters, and Blood Collection

Subject recruitment was conducted at WesternU’s on-campus clinic. Once recruited, a pre-screen was conducted on each subject before enrollment and randomization of each participant into either the experimental or placebo group. The pre-screen appointment was conducted with the appointed physician for the study and included a comprehensive metabolic panel, testing for HbA1c (Glycated Hemoglobin) levels, hepatitis B surface antigens, HIV antibody screening and if applicable, pregnancy.

The inclusion and exclusion criteria were identical for both the experimental and placebo groups of the clinical trial. Inclusion criteria for subject enrollment, included subjects aged: 18-65 years old, positive for T2DM, had HbA1c levels between 7-13%, negative for HIV, and no history of latent tuberculosis infection or liver function abnormalities. Previous studies have indicated that recurrent alcohol abuse in addition to past hepatitis infections may impair liver function while independently causing depletion of GSH levels [26]. Therefore, the exclusion criteria included: positive for HIV and evidence or history of abnormal liver function due to either prolonged alcohol abuse or chronic viral hepatitis. Alcohol abuse was determined using an AUDIT (Alcohol Use Disorders Identification Test) questionnaire, with scores >8 indicative of unhealthy alcohol abuse, resulting in omission from the study [27]. In addition to the pre-screening laboratory tests, the pre-screen appointment consisted of a physical examination, vital measurements, and a comprehensive review of symptoms and medications. After each potential candidate was cleared for enrollment by the study’s appointed physician, the subject was randomized into either the experimental or placebo group. Although our inclusion criteria included participants age between 18 to 65 years old, the enrolled subjects of our study had a mean age of 54.86, a median age of 52, with the youngest and oldest subject age of 48 and 65 years old, respectively. After enrollment, three additional in-person visits were scheduled: Visit #1 (Baseline), Visit #2 (Midway point), Visit #3 (Post-Treatment). The pre-screening appointment, Visit #1, and Visit #3 all required a 50 mL venous blood draw, while Visit #2 was a compliance check. Subjects were all compensated for their involvement in the study.

### Isolation and Storage of Peripheral Blood Mononuclear Cells

Whole blood samples underwent density gradient centrifugation using ficoll histopaque (Sigma, St. Louis, MO, USA), which involved layering whole blood samples onto ficoll histopaque in a 1:1 ratio, centrifuging at 25°C for 30 minutes at 800 G, and using a slow brake to prevent disturbance of the gradient. This allowed for isolation of Peripheral Blood Mononuclear Cells (PBMCs). PBMCs were washed twice with a 1x phosphate-buffered saline (PBS) solution and suspended in 1mL cold fetal bovine serum (FBS) for cryopreservation. FBS 20% DMSO (Dimethyl Sulfoxide) was then slowly added to the suspension, until a final concentration of FBS 10% DMSO was reached. This sample was aliquoted into separate vials to be stored at −80°C prior to *in vitro* infection.

### Thawing Cryopreserved PBMCs

Cryopreserved PBMCs in FBS 10% DSMO were partially thawed in a warm water bath heated to 37°C. Partially thawed PBMCs were then washed 2x with warm RPMI (Sigma, St. Louis, MO, USA) with 5% human AB serum (Sigma, St Louis, MO, USA) and centrifuged at 600 G for 20 mins at 25°C. PBMCs were resuspended in warm RPMI with 5% human AB serum, and then distributed at 6 x 10^5^ cells/well onto 24-well plates (Corning, NY, USA) coated with .001% poly-lysine (Sigma, St. Louis, MO, USA). PBMC cell counts were determined with trypsin blue exclusion staining. PBMCs were incubated overnight in a 24-well plate at 37°C with 5% CO_2_ prior to *in vitro* infection.

### *In vitro* PBMC Infection and Treatment

PBMCs were infected with *M. bovis* BCG (BCG) strain at a multiplicity of infection (MOI) of 0.1:1 cell ratio following overnight incubation. PBMCs were then, either sham-treated (untreated) or treated with the one-time addition of 1 nM of everolimus on the same day of infection. Infected and treated PBMCs were maintained at 37°C, with 5% CO_2_ until termination at 8- or 15-days post-infection.

### Terminating *in vitro* Granulomas to Determine Survival of BCG

Granulomas serve as the main host defense mechanism against mycobacterium dissemination by forming a cellular barrier around the pathogen. Various immune cells, including macrophages, dendritic cells, epithelioid histiocytes, T-cell, fibroblasts, giant cells, and natural killer cells form the granuloma approximately seven days post-BCG infection [12,28]. Therefore, infected PBMCS were terminated at 8- and 15- days post-infection. Granulomas generated *in vitro* were harvested to determine the survival of BCG. Cell-free supernatants were collected and stored at −20°C and granulomas were harvested by adding ice-cold sterile 1x PBS to wells, followed by gentle scraping to achieve maximum recovery of granuloma lysates and stored at −20°C. Granulomas were vortexed followed by freeze/thaw cycles to lyse cells and release intracellular BCG. Cell supernatants and lysates were plated on 7H11 agar medium (Hi Media, Santa Maria, CA, USA) enriched with ADC (GEMINI, USA) and incubated at 37°C for three weeks to evaluate mycobacterial growth or survival under treatment conditions by counting the colony-forming units (CFUs). CFUs were corrected for any dilutions made to the supernatant or lysates before plating.

### Cytokine Measurements

In order to determine the effects of *in vitro* everolimus treatment and *in vivo* GSH in altering the levels of certain cytokines, measurements of IFN-γ, TNF-α, IF-6, and IF-10 were performed in the supernatants from the granulomas. Enzyme-linked immunosorbent (ELISA) assay kits were utilized for quantification of cytokine levels including Human IL-10 ELISA Ready-SET-Go from Affymetrix (Cat# 88-7106) and Human TNF-α Uncoated ELISA (Cat # 88-7346), Human IFN-γ Uncoated ELISA (Cat # 88-7316), and Human IF-6 Uncoated ELISA (Cat #88-7066) from Invitrogen. The manufacturer’s protocol in each kit was utilized for all assays.

### Statistical Analysis and Sample Size

The GraphPad Prism Software 8 was utilized for statistical analysis. One-way ANOVA (Analysis of Variance) was performed when comparing more than two categorical variables with Tukey corrections applied. The unpaired T-test was performed when comparing only two categories with Welch corrections applied. All values reported are representative of the mean values for each respective category and a p-value of < 0.05 was considered significant. Any placement of an asterisk (*) denotes a direct comparison to the previous category. Any placement of a hash mark (#) signifies the comparison between a category and the previous category that is exactly a single column before the marked column. Any placement of a dollar sign ($) denotes the comparison between a category and the previous category that is exactly two columns before the marked column. When two symbols are represented (**, ##, or $$), a significant difference below 0.005 is meant to be implied. The clinical trials sample size included eleven T2DM positive subjects in the treatment (L-GSH supplementation) arm and seven T2DM positive subjects in the control (placebo) arm.

## Results

### Clinical Trial Findings for *In Vitro* Everolimus Studies: Intracellular Survival of BCG within *n vitro* Granulomas of T2DM Subjects

**Figure 2:**
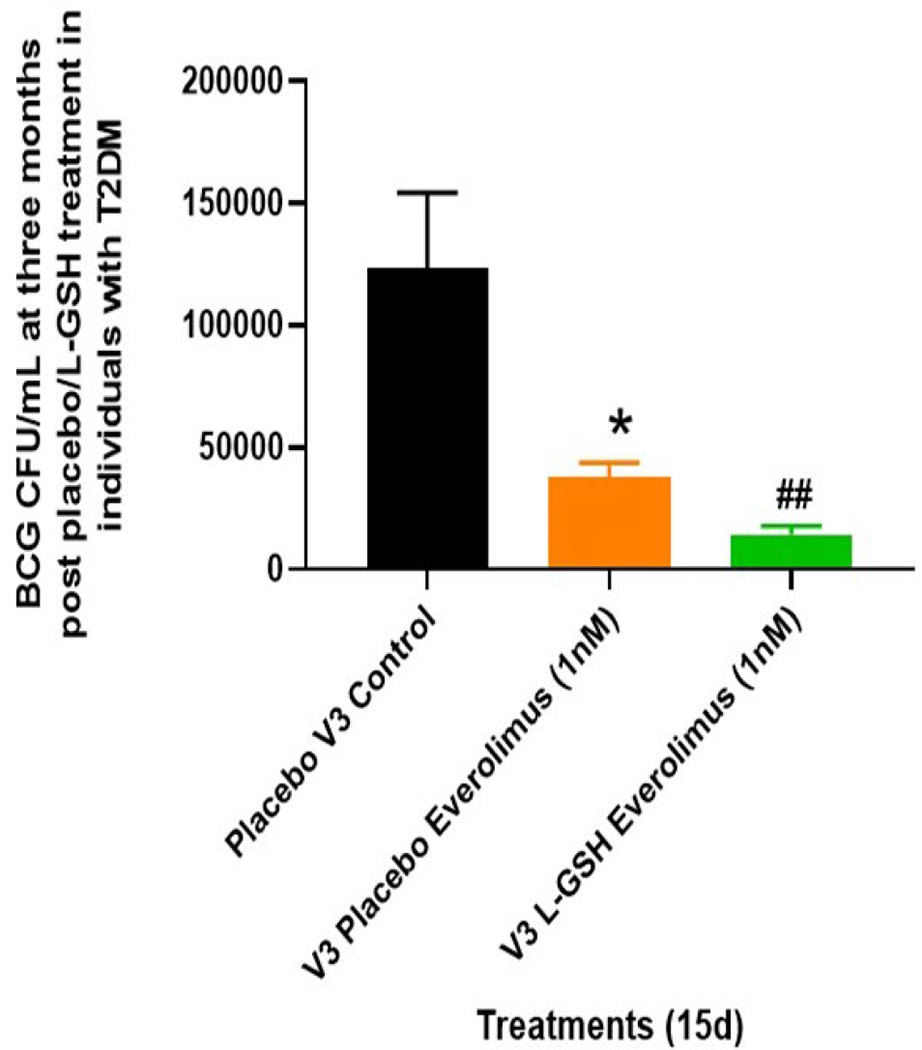
Clinical trial findings from *in vivo* and *in vitro* studies. Determination of BCG survival in the *in vitro* granulomas of T2DM subjects on L-GSH/ or placebo supplement. PBMCs isolated from individuals with type 2 diabetes at three months post- L-GSH/ or placebo supplementation were infected *in vitro* with BCG and treated *in vitro* with everolimus (1nM) and terminated at 15 days post-infection. The survival of BCG in the *in vitro* granulomas was determined by plating the granuloma lysates on 7H11 agar plates containing ADC. V3 represents intracellular survival of BCG within *in vitro* granulomas generated from PBMCs isolated from subjects with T2DM at three months post-L-GSH/ or placebo supplementation. Placebo control represents samples from T2DM subjects on placebo supplementation who’s infected-PBMCs did not receive *in vitro* everolimus treatment. The categories labeled with EV represent samples from the L-GSH or placebo groups that were treated with *in vitro* everolimus. The GraphPad Prism Software 8 was utilized for statistical analysis. One-way ANOVA was performed when comparing more than two categorical variables with Tukey corrections applied. All values reported are representative of the mean values for each respective category and a p-value of < 0.05 was considered significant. Any placement of an asterisk (*) denotes a direct comparison to the previous category. Any placement of a hash mark (#) signifies the comparison between a category and the previous category that is exactly a single column before the marked column. When two symbols are represented (** or ##), a significant difference below 0.005 is meant to be implied. The clinical trials sample size included eleven T2DM positive subjects in the treatment (L-GSH supplementation) arm and seven T2DM positive subjects in the control (placebo) arm.

Intracellular survival of BCG within *in vitro* granulomas generated from PBMCs isolated from T2DM subjects was measured in both he L-GSH and placebo groups at pre- and post- supplementation Infected-PBMCs were either untreated (control) or treated with a one-time *in vitro* addition of 1ng/ml everolimus. When compared to untreated (control) PBMCs, *in vitro* everolimus treatment resulted in a 50% reduction in the viability of BCG (Figure 2). In addition, compared to the placebo group with everolimus treatment, the L-GSH group with everolimus treatment, had a further significant reduction of the intracellular viability of BCG of over 50% (Figure 2). These results indicate that *in vitro* everolimus treatment was able to significantly reduce the intracellular viability of BCG. When everolimus was coupled with a three-month clinical intervention of L-GSH, it produced a further significant reduction in BCG viability and significantly improved the control of *in vitro* BCG infection within treated granulomas compared to the placebo control (untreated) group.

### Clinical Trial Findings for *In Vitro* Everolimus Studies: Quantification of Cytokines Levels within Granuloma Supernatants of PBMCs Infected *In Vitro* with BCG in T2DM Subjects

**Figure 3:**
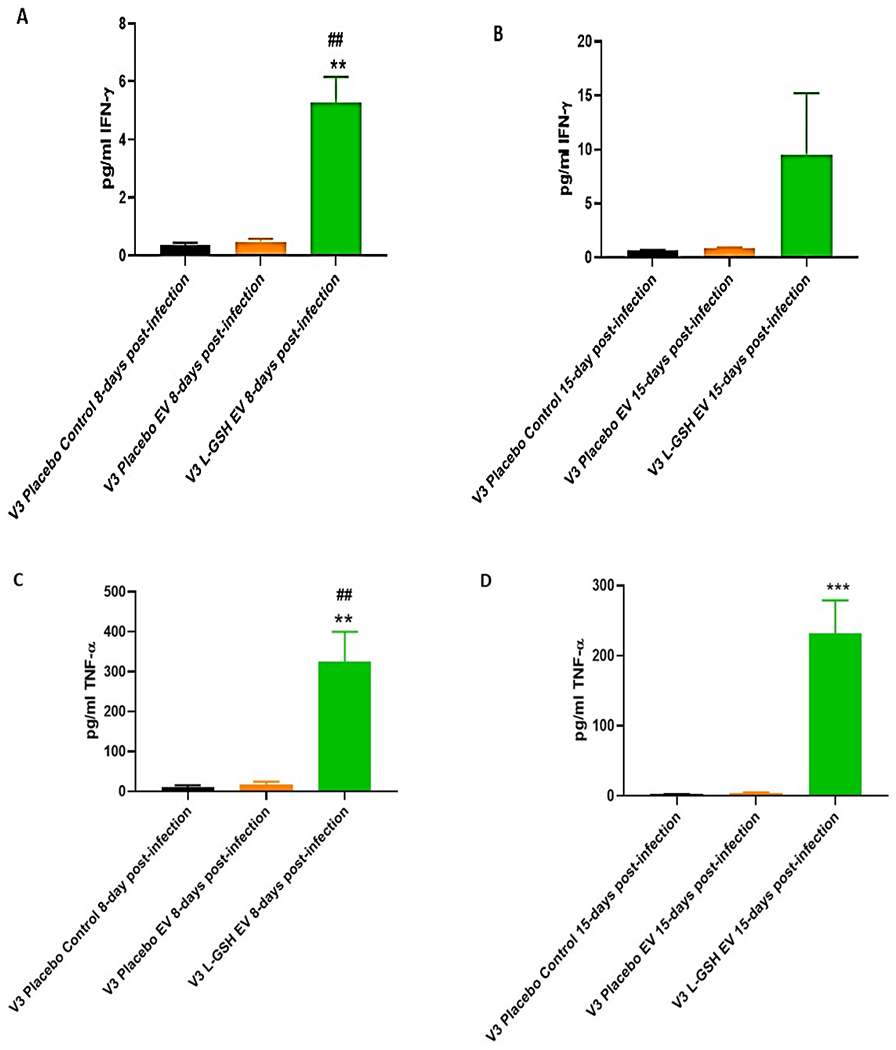

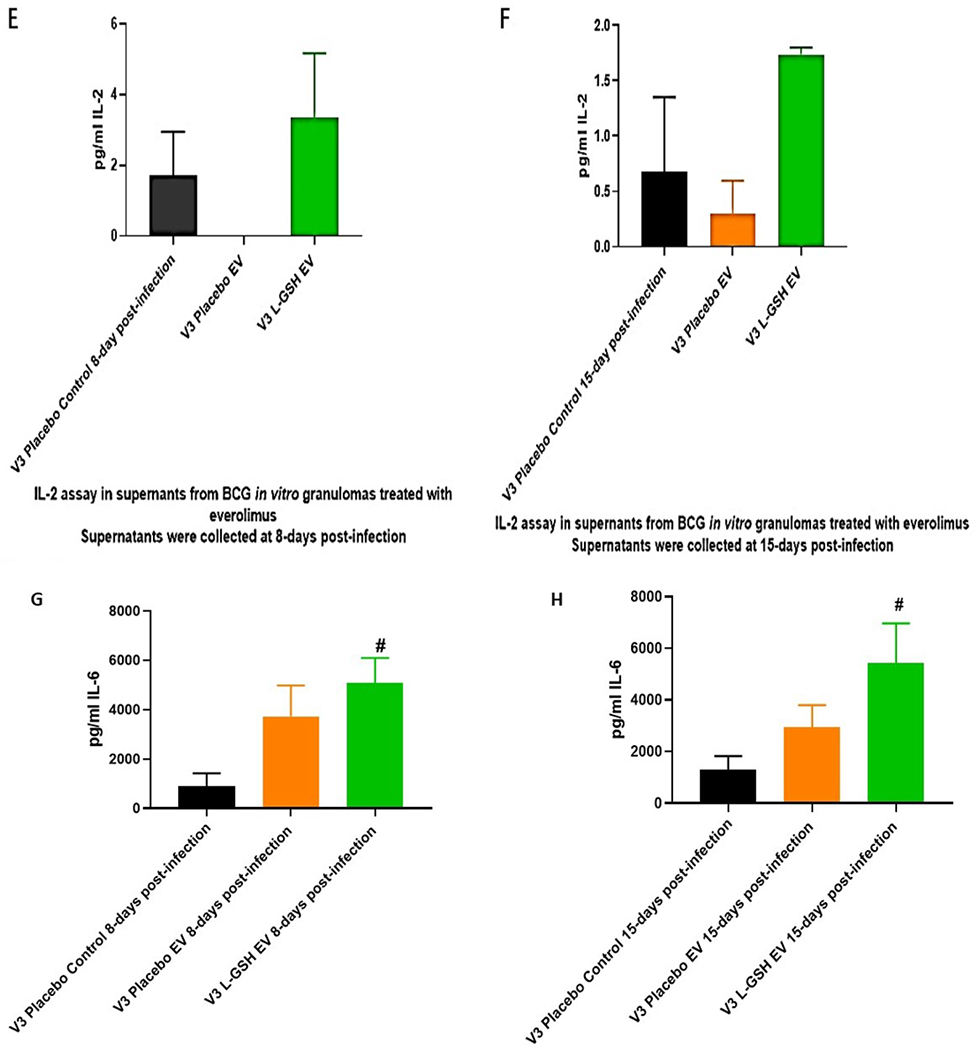
**A, B, C, D, E, F, G, H:** Clinical trial findings from *in vivo* and *in vitro* studies. Quantification of IFN-γ, TNF-α, IL-2 and IL-6 in the *in vitro* granulomas of T2DM subjects on L-GSH/or placebo supplement. PBMCs isolated from individuals with type 2 diabetes at pre- and post- L-GSH/or placebo supplementation were infected *in vitro* with BCG and treated *in vitro* with everolimus (1nM) and terminated at 8 days (Figure 3A, C, E, G) or 15 days (Figure 3B, D, F, H) post-infection. Levels of IFN-γ (Figure 2A, B), TNF-α (Figure 3C, D), IL-2 (Figure 3E, F) and IL-6 (Figure 3G, H) in the granuloma supernatants were determined by ELISA using an assay kit from Invitrogen. V3 represents *in vitro* granulomas supernatant samples generated from PBMCs isolated from subjects with T2DM at three months post- L-GSH/ or placebo supplementation. Placebo control represents samples from T2DM subjects on placebo supplementation who’s infected-PBMCs did not receive *in vitro* everolimus treatment. The categories labeled with EV represent samples from the L-GSH or placebo groups that were treated with *in vitro* everolimus. The GraphPad Prism Software 8 was utilized for statistical analysis. One-way ANOVA was performed when comparing more than two categorical variables with Tukey corrections applied. All values reported are representative of the mean values for each respective category and a p-value of < 0.05 was considered significant. Any placement of an asterisk (*) denotes a direct comparison to the previous category. Any placement of a hash mark (#) signifies the comparison between a category and the previous category that is exactly a single column before the marked column. When two symbols are represented (** or ##), a significant difference below 0.005 is meant to be implied. The clinical trials sample size included eleven T2DM positive subjects in the treatment (L-GSH supplementation) arm and seven T2DM positive subjects in the control (placebo) arm.

Cytokine levels were quantified from *in vitro* granulomas supernatants that were generated from PBMCs isolated from T2DM subjects in both the L-GSH and placebo groups at pre- and post- supplementation. Infected-PBMCs were either untreated (control) or treated with a one-time *in vitro* addition of 1ng/ml everolimus. A statistically significant increase in IFN-γ (Figure 3A, B), TNF-α (Figure 3C, D), IL-2 (Figure 3 E, F), and a notable increase of IL-6 (Figure 3G, H) was observed in the everolimus treated L-GSH supplemented granuloma supernatant at both 8- and 15- days post *in vitro* BCG infection, when compared to the placebo control (untreated) groups (Figure 3A, B, C, D, E, F, and G). These results indicate that a three-month clinical intervention of L-GSH coupled with everolimus treatment significantly increased levels of IFN-γ, TNF-α, and IL-2 in response to *BCG* infection when compared to the placebo control (untreated) groups.

## Discussion

Individuals with T2DM have lowered levels of GSH compared to healthy subjects due to decreased levels of the GSH metabolism enzymes: γ-glutamyl cysteinyl ligase (GCL) and glutathione synthetase (GSS) [29]. In the same study, increased levels of transforming growth factor-beta (TGF-β), a cytokine known to reduce the expression of GCL, were observed in plasma samples from T2DM subjects when compared to the healthy group [29]. These lowered levels of GSH and the GSH metabolism enzymes in T2DM individuals led to increased susceptibility to an *M. tb.* Infection [29]. Our lab has also shown that GSH deficiency is correlated with increased levels of free radicals and intracellular *M. tb.* viability [30,31]. Additionally, our lab has found that GSH has direct antimycobacterial activity, thus functioning as an effector molecule in the innate defense against *M. tb.* [32]. *In vitro* restoration of GSH with the glutathione precursor: N-Acetyl Cysteine (NAC), combined with antibiotics, INH and RIF, resulted in bolstering cytokine modulation and complete clearance of *M. tb.* infection in granulomas generated from PBMCs of healthy individuals and significant reduction in *M. tb* burden in individuals with T2DM [26].

In another previous preclinical study, we tested the effects of everolimus, alone and in combination with current first-line antibiotics (isoniazid and pyrazinamide), added at the minimum inhibitory concentration (MIC), against an *M. tb* infection within *in vitro* human granulomas [22]. We found that *M. tb*-infected *in vitro* granulomas treated with everolimus alone resulted in significantly decreased *M. tb* burden compared to similar granulomas in the control group [22]. Cells treated with everolimus doses of either 1 nM or 2 nM in conjunction with pyrazinamide (PZA) produced a significant reduction in the intracellular *M. tb* burden. Treatment groups that received everolimus also experienced a significant reduction in oxidative stress, and higher levels of autophagy and mTOR inhibition. Results from this study indicate that everolimus is efficacious in controlling *M. tb* infection in the granulomas and has additive effects when combined with isoniazid and pyrazinamide [22]. This study demonstrated everolimus to be a promising HDT in the context of *M. tb* infection within the *in vitro* granuloma model. Further study is warranted to better characterize these effects.

In this clinical trial study, individuals with T2DM were either supplemented with oral L-GSH or placebo supplementation for three months. At pre- and post-supplementation for both the L-GSH and placebo group, immune cells were isolated from the T2DM subjects for conducting *in vitro* assays with everolimus to determine its efficacy alongside *in vivo* L-GSH on improving control of an *in vitro* BCG infection within *in vitro* granulomas (Figure 1 and Table I).

L-GSH supplementation for three months along with *in vitro* everolimus demonstrated to have improved the control of an *in vitro* BCG infection within *in vitro* granulomas. Compared to the placebo group, the L-GSH group had significantly less burden of BCG even when both groups were treated with *in vitro* everolimus (Figure 2). L-GSH supplementation was also able to modulate cytokines levels of IFN-γ, TNF-α, and IL-2 within *in vitro* granulomas treated with everolimus. When compared to the placebo group, L-GSH supplementation was able to significantly increase levels of IFN-γ (Figure 3A, B), TNF-α (Figure 3C, D), and IL-2 (Figure 3 E, F) levels found within supernatants of *in vitro* granulomas treated with everolimus in T2DM subjects. These findings of increased levels of IFN-γ, TNF-α, and IL-2 (Figure 3) in the granuloma supernatants, alongside the findings that *in vitro* everolimus in combination with L-GSH supplementation was able to further decrease intracellular BCG viability (Figure 2) demonstrates the efficacy of using of everolimus in combination with L-GSH supplementation to enhance the control of a BCG infection within *in vitro* granulomas of individuals with T2DM.

As everolimus is an mTOR inhibitor known to upregulate autophagy, the increased ability to control infection signifies the ability for everolimus to modulate the immune system through the mTOR pathway. In our previous study mentioned earlier, we did observe decreased levels of mTOR expression and increased levels of LC3B expression, an autophagy marker, within *in vitro* granulomas treated with everolimus at 1 nM [22]. More studies should be conducted to establish the reduction in the mycobacterial burden with the upregulation of autophagy through mTOR inhibition.

In conclusion, our studies have demonstrated that everolimus at 1 nM produced immune-enhancing effects to more effectively reduce the mycobacterial burden when used in combination with *in vivo* supplementation with L-GSH in individuals with T2DM than alone. However, *in vitro* everolimus alone at 1 nM still improved mycobacterial burden, but not to the same extent as with *in vivo* supplementation with L-GSH. This improved ability to control intracellular mycobacteria could be through the upregulation of autophagy by inhibition of mTOR by everolimus and through the restoration of redox homeostasis by the L-GSH in the cell to effectively facilitate a more effective Th1 response, as seen in the increased levels of IFN-γ, TNF-α, and IL-2. Therefore, everolimus coupled with L-GSH supplementation in individuals with T2DM can improve the control of mycobacterial infections.

As the prevalence of T2DM increases globally and is recognized as a reemerging risk and challenge to the management of TB, oral L-GSH could be utilized as adjunct therapy alongside current anti-TB treatments or even as a preventative strategy for individuals with T2DM with known dysregulation of GSH metabolism and intracellular redox balance. Everolimus can also be employed alongside the current anti-TB drugs to reduce treatment duration and adverse side effects and as a potential HDT that can prevent MDR/XDR-TB emergence. If L-GSH and everolimus are used in combination it could further help enhance the current anti-TB treatments to manage TB.

## Figures and Tables

**Table 1:** *In Vivo* vs. *In Vitro* Study Components. The *in vivo* components of this study include the clinical intervention of either oral liposomal glutathione (L-GSH) or the placebo (empty liposomes) supplementation for three months in the T2DM subjects. Blood was drawn from study subjects in each the L-GSH and placebo arm to conduct *in vitro* studies on isolated PBMCS from the whole blood. The *in vitro* study components include an *in vitro* infection with *M. bovis* BCG strain and a one-time addition of everolimus at 1nM, or an untreated control.

In Vivo	In Vitro
Oral Supplementation	Infection
• **Liposomal Glutathione (L-GSH)** for 3 months	• ***Mycobacterium Bovis (M. bovis)*** BCGH
Oral Supplementation	Treatments
• **Empty Liposomes (Placebo)** for 3 months	• **Control (no treatment)** • **Everolimus**

## Data Availability

The datasets generated during and/or analysed during the current study are available from the corresponding author on reasonable request.
